# To what extent does sociodemographic composition of the neighbourhood explain regional differences in demand of primary out-of-hours care: a multilevel study

**DOI:** 10.1186/s12875-015-0275-0

**Published:** 2015-05-06

**Authors:** Tessa Jansen, Marieke Zwaanswijk, Karin Hek, Dinny de Bakker

**Affiliations:** NIVEL, Netherlands Institute for Health Services Research, P.O. Box 1568, 3500 BN Utrecht, The Netherlands; Department of Social and behavioural science, Scientific Centre for Transformation in Care and Welfare (TRANZO), Tilburg University, PO Box 90153, 5000 LE Tilburg, The Netherlands

**Keywords:** Primary out-of-hours care, GP cooperative, Sociodemographic composition, Neighbourhood, Multilevel linear regression

## Abstract

**Background:**

In the Netherlands, primary out-of-hours (OOH) care is provided by large scale General Practitioner (GP) cooperatives. GP cooperatives can be contacted by patients living in the area surrounding the GP cooperative (catchment area) at hours when the patient’s own general practice is closed. The frequency of primary OOH care use substantially differs between GP cooperative catchment areas. To enable a better match between supply and demand of OOH services, understanding of the factors associated with primary OOH care use is essential. The present study evaluated the contribution of sociodemographic composition of the neighbourhood in explaining differences in primary OOH care use between GP cooperative catchment areas.

**Methods:**

Data about patients’ contacts with primary OOH services (n = 1,668,047) were derived from routine electronic health records of 21 GP cooperatives participating in the NIVEL Primary Care Database in 2012. The study sample is representative for the Dutch population (for age and gender). Data were matched with sociodemographic characteristics (e.g. gender, age, low-income status, degree of urbanisation) on postcode level. Multilevel linear regression models included postcode level (first level), nested within GP cooperative catchment areas (second level). We investigated whether contacts in primary OOH care were associated with neighbourhood sociodemographic characteristics.

**Results:**

The demand of primary OOH care was significantly higher in neighbourhoods with more women, low-income households, non-Western immigrants, neighbourhoods with a higher degree of urbanisation, and low neighbourhood socioeconomic status. Conversely, lower demand was associated with neighbourhoods with more 5 to 24 year old inhabitants. Sociodemographic neighbourhood characteristics explained a large part of the variation between GP cooperatives (R-squared ranging from 8% to 52%). Nevertheless, the multilevel models also showed that a considerable amount of variation in demand between GP cooperatives remained unexplained by sociodemographic characteristics, particularly regarding high-urgency contacts.

**Conclusions:**

Although part of the variation between GP cooperatives could not be attributed to neighbourhood characteristics, the sociodemographic composition of the neighbourhood is a fair predictor of the demand of primary OOH care. Accordingly, this study provides a useful starting point for an improved planning of the supply of primary OOH care.

## Background

The provision of appropriate and timely out-of-hours (OOH) care is central for efficient and effective use of the healthcare system [1-3]. In most Western health care systems, OOH care is provided by two organisations [4]: (1) hospital emergency departments (EDs), which generally handle highly urgent health problems; and (2) primary care out-of-hours services, which are intended for less urgent health problems that cannot wait until office hours [5]. Primary OOH services provide care between 5 pm and 8 am, in weekends and on national holidays. Overuse of primary OOH care is a major issue, resulting in inefficient use of the health care system and high workload of care providers [5,6]. Although patients contacting primary OOH services may perceive their problem as urgent, the majority of patients contact primary OOH services for low-urgency health problems [4], and for problems that can wait until the next day [5]. Therefore, to address issues of appropriate and inappropriate use, insight in actual utilisation is essential. Ideally, actual utilisation is determined by factors associated with the needs of the population. Consequently, understanding of the population factors influencing the use of primary OOH care is necessary the be able to analyse over and underuse [2,7].

In the past years, in several European countries the organisation of primary OOH care has changed from small-scale rota-groups to large-scale organisation models [5]. The present study focuses on the demand of primary OOH care in the Netherlands. Similar to health care systems in Denmark and the United Kingdom [5], Dutch primary OOH care is provided by large general practitioner cooperatives (GP cooperatives). The GP cooperative as organisation model for primary OOH care, was found to have many strengths, e.g. efficiency and reduction of the GPs’ workload, compared to different primary OOH care models in Europe [2]. GP cooperatives in the Netherlands are networks of, on average, 144 affiliated general practitioners (GPs) [8], providing OOH care for 50,000 to 500,000 patients [9] in specified postcode areas surrounding the GP cooperative (the catchment area). Currently, in the Netherlands, 55 GP cooperatives provide primary OOH care at 128 locations [8]. Patients can receive primary OOH care via telephone consultations, consultations at the cooperative, and home visits [3,10]. In 2012, GP cooperatives on average claimed 233 contacts per 1000 inhabitants of their catchment area [11]. However, large regional variation existed between GP cooperatives in the number and nature of the claimed contacts [8].

Sociodemographic characteristics of individual patients, such as age and gender, have been found to affect the demand of primary OOH care [2,12]. The relationship between neighbourhood population characteristics and health outcomes has been studied extensively, and indicated independent neighbourhood effects, in addition to individual effects [13]. Moreover, neighbourhood population characteristics were found to affect health care use. For instance, physician use was found to be related to both individual characteristics, and to neighbourhood deprivation independent from individual characteristics [7]. Associations between individual characteristics and health care use may be amplified or reduced at the aggregated neighbourhood level. For example, in deprived neighbourhoods, social networks may be weak and affect adverse health outcomes [14], resulting in more health care utilisation [15]. Additionally, in neighbourhoods with a large elderly population, availability of informal care may be insufficient, and therefore increase the demand of formal health care [16,17]. Accordingly, the aim of the present study was to evaluate the extent to which neighbourhood sociodemographic composition (e.g. gender, age and low-income status of the population, degree of urbanisation), contributes to explaining differences in the demand of primary OOH care between GP cooperative catchment areas.

## Methods

### Data

Data of primary OOH contacts in 2012 (1/1/2012 – 31/12/2012) were derived from routine electronic health records of 21 GP cooperatives participating in the NIVEL Primary Care Database [11] (http://www.nivel.nl/en/dossier/nivel-primary-care-database). This database contains unidentifiable patient records that are routinely collected from primary health care providers.

The population in the catchment areas of the included GP cooperatives is representative of the Dutch population with regard to gender and age. All GP cooperatives registered patient records for at least 46 weeks during 2012. Demand of primary OOH care was calculated based on all claimed contacts. For each contact with the primary OOH service the following data were available: four-digit postcode, urgency of the presented health problem, and symptoms and diagnoses presented during the contact (see Measures).

Some postcode areas were covered by more than one GP cooperative catchment area. These were omitted from the analyses. The number of contacts with the primary OOH service was summed for each patient, and aggregated to four-digit postcode level. Subsequently, sociodemographic characteristics of the postcode area were matched. Postcode areas with at least 1000 inhabitants and for which data for at least 100 patients were available, were included in the analyses. Estimates based on small samples may induce bias due to a large relative effect of a small sub sample resulting from local variations [18]. Consequently, models based on areas with less than 1000 inhabitants and less than 100 patients were not considered reliable [19]. This resulted in 1121 postcode areas that were eligible for analyses, covering 23% of the postcode areas in the Netherlands. With 7.3 million inhabitants, nearly 44% of the Dutch population was included. Records of 1,112,508 patients (1,668,047 contacts) were available for analyses.

### Measures

#### Outcomes

From the electronic health records, the following outcome measures were derived and calculated per 1000 inhabitants:

Contacts included total number of contacts, number of telephone consultations, consultations, and home visits.

Urgency of contact was derived from the urgency level assigned by a triage assistant. Patients must seek contact with the GP cooperative by telephone before attending. Trained assistants (under supervision of a GP) execute the telephone triage by using a standardised six-level triage system, the Netherlands Triage System (NTS) [20]. Urgency levels were dichotomised in high urgency (U0: resuscitation, U1: life-threatening, U2: emergency, and U3: urgent), and low urgency (U4: non-urgent, and U5: self-care advice).

Symptoms and diagnoses presented during primary OOH contacts are registered by ICPC-codes from the International Classification of Primary Care version 1, as are used by GPs in the Netherlands [21,22]. Individual ICPC-codes were grouped in five disease clusters (Table 1), containing the majority (70%) of out-of-hours contacts. In the analyses including ICPC-codes, only data were used of GP cooperatives of which at least 70% of the contacts included an ICPC-code that could be categorised into a disease cluster. ICPC-codes that were categorised, range from 01 to 29 (symptoms), and from 70 to 99 (diagnoses). ICPC-codes A97 (no disease) and A99 (other generalised disease/multiple syndromes) are sometimes used when health care providers do not directly know what is wrong with a patient. However, they cannot be categorised in a disease cluster, and therefore, we did not include these ICPC-codes in the analyses. The same holds for codes in the range 30–69 (procedures). Consequently, data from 14 GP cooperatives, covering 620 postcode areas, were included in the analysis of contacts by disease cluster. Some contacts included more than one ICPC-code. Since the measurement unit was the number of contacts, and counting more ICPC-codes per contact as single contact would have inflated the number of contacts, fractions were calculated, i.e., when three ICPC-codes for one contact were registered, the contact counted as one third of a contact for each ICPC-code.

**Table 1:** **Examples of symptoms and diagnoses in each disease cluster**

**Disease cluster**	**Examples of symptoms and diagnoses**
Injuries	Laceration/ cut; bruise/ contusion; burn/ scald; animal/ human bite; foreign body in eye; sprain/ stain of knee.
Infections	Cystitis/ urinary infection other; acute otitis media/ myringitis; pneumonia; gastroenteritis presumed infection.
Long-term health conditions	COPD; asthma; diabetes mellitus; incontinence urine; migraine; malignant neoplasm bronchus/ lung; constipation.
Psychological and social problems	Concern/ fear medical treatment; acute stress reaction; depressive disorder; acute alcohol abuse.
Somatic symptoms and illnesses	Fever; cough; chest symptom complaint; abdominal pain/ cramps general; fainting syncope; nausea.

#### Independent variables

Sociodemographic characteristics of the neighbourhood, on four-digit postcode level, were obtained from census records of Statistics Netherlands [20]: number of inhabitants of the area, number of male and female inhabitants in age categories, number of low-income households (yearly purchasing power of less than € 9,250), number of non-Western immigrants (at least one parent born in Africa, Latin America, Asia excluding Indonesia and Japan, or Turkey), and degree of urbanisation in five categories (from rural: fewer than 500 addresses/km^2^, to very strongly urbanised: 2500 or more addresses/km^2^). In addition, neighbourhood status scores, on four-digit postcode level, were derived from The Netherlands Institute for Social Research (SCP) [23]. This score reflects the social status of a neighbourhood, compared to other neighbourhoods in the Netherlands. It is a composite measure calculated from individual characteristics of neighbourhood inhabitants, i.e. mean neighbourhood income, percentage of residents with low-income, percentage of low-educated residents, and percentage of residents without a job. Factor analysis was conducted to compile these characteristics to a single indicator [23]. Status scores were categorised in quartiles [24] (low, moderate, high, and very high status). Status score is a common indicator for neighbourhood socioeconomic status (SES) in the Netherlands [25].

Percentages per postcode were calculated for the following sociodemographic factors: females, age group 0 to 4 years, age group 5 to 14 years, age group 15 to 24 years, age group 25 to 39 years, age group 40 to 64 years, age group 65 to 74 years, age group 75 years and older, non-Western immigrants, low-income households, and single-person households. Urbanisation variables and neighbourhood status scores were dummy-coded.

### Statistical analyses

Analyses were conducted using Stata version 13.1 (StataCorp LP). First, descriptive analyses were conducted to describe the study population and the distribution of the outcome variables.

Subsequently, to analyse whether sociodemographic characteristics were related to contacts in primary OOH services, multilevel linear regression analyses were conducted. Initially, percentage of single-person households was included in the analyses, however due to high multicollinearity, this variable was omitted. Although the percentage of persons with low income partly constitutes the neighbourhood status score, no multicollinearity was found for this variable.

To account for clustering of contact data within GP cooperative catchment areas, multilevel linear regression models were used for all analyses [26,27]. Multilevel models included four-digit postcode areas (first level), nested within GP cooperative catchment areas (second level), using the restricted maximum likelihood (REML) method. Explained variance of the multilevel models, R-squared, were calculated using the method as prescribed by Snijders and Bosker [28]. Associations were considered significant if p-values were < .001.

### Privacy

Dutch law allows the use of extracts of electronic health records for research purposes under certain conditions. According to Dutch legislation, neither obtaining informed consent nor approval by a medical ethics committee is obligatory for this kind of observational studies (Dutch Civil Law, Article 7:458; http://www.dutchcivillaw.com/civilcodebook077.htm). Nevertheless, GP cooperatives participating in the NIVEL Primary Care Database are contractually obliged to inform patients about the GP cooperatives’ participation in the NIVEL Primary Care Database, and to inform patients about the possibility to opt-out if they objected to their data being included in the database. For more detail: [29]

## Results

### Sample characteristics and primary OOH contacts

Sociodemographic characteristics of the sample are depicted in Table 2. Table 3 shows the number of contacts with a GP cooperative per 1000 inhabitants. Consultations at the GP cooperative were the most frequently occurring type of contact, slightly more contacts are of low urgency than of high urgency, and most symptoms and diagnoses presented are part of the cluster ‘somatic symptoms and illnesses’.

**Table 2:** **Sociodemographic characteristics of the sample** ^**a**^ **, and comparison with the Dutch population (2012)**

	**Mean (SD)**	**Sample**	**Dutch population**
	n	%	%
Inhabitants (total n = 7,269,160)	6485 (4156.9)		
Patients (total n = 1,112,508)	992 (674.5)		
Female		50.2	50.5
Age group 0–4 years		5.5	5.5
5-14 years		11.8	11.9
15-24 years		12.3	12.3
25-39 years		18.7	18.8
40-64 years		36.1	35.9
65-74 years		8.6	8.6
75+ years		7.0	7.0
Non-Western immigrants		11.0	11.0
Low-income households		40.7	40.0
Urbanisation Rural		28.3	-
Low urbanisation		18.7	-
Moderate urbanisation		15.2	-
High urbanisation		17.8	-
Very high urbanisation		20.0	-
Neighbourhood status Low status		31.9	-
Moderate status		23.2	-
High status		23.6	-
Very high status		21.4	-

^a^Four-digit postcode areas with > = 1000 inhabitants and > = 100 patients (n = 1121).

**Table 3:** **Mean number of primary out-of-hours contacts per 1000 inhabitants for 2012** ^**a**^

	**Mean number of contacts (SD) (per 1000 inhabitants)**
Total number of contacts with GP cooperative: 1,668,047	
All contacts	224 (56.4)
Telephone consultations	92 (25.2)
Home visits	23 (12.0)
Consultations	109 (32.3)
Urgency of contacts High	106 (35.6)
Low	118 (36.8)
Disease cluster^b^ Injuries	27 (13.8)
Infections	34 (16.4)
Long-term health conditions	23 (13.5)
Somatic symptoms and illnesses	64 (36.2)
Psychological and social problems	8 (6.3)

^a^Per 1000 inhabitants per four-digit postcode area with > = 1000 inhabitants and > = 100 patients (n = 1121).

^b^Contacts per disease cluster are calculated using data of 14 GP cooperatives that registered meaningful ICPC-codes for at least 70% of contacts, resulting in data for 619 postcode areas. Numbers do not add up to total number of contacts because we included a selection of symptoms and diagnoses.

### Multilevel linear regression analyses

Results of multilevel linear regression analyses are depicted in Tables 4 (for type and urgency of OOH contacts) and 5 (OOH contacts per disease cluster). Most neighbourhood sociodemographic characteristics were significantly associated with the total number of contacts with a GP cooperative. Neighbourhoods with more women, more low-income households, more non-Western immigrants, a higher degree of urbanisation, and lower neighbourhood status were associated with more primary OOH contacts. The degree of urbanisation showed a positive association, with a higher number of primary OOH contacts in increasing levels of urbanisation. Except, however, for the most urbanised neighbourhoods, which showed a decrease in the number of contacts. In neighbourhoods that inhabited more children of 5 to 14 years old, and adolescents (15 to 24 years old) there were less primary OOH contacts. For telephone consultations and consultations, a similar pattern was observed, although in neighbourhoods with more 0 to 4 year old children, more (telephone) consultations took place. Conversely, home visits were mainly associated with more elderly inhabitants (≥75 years) in a neighbourhood. Associations between sociodemographic characteristics and urgency of the primary OOH contact, followed parallel patterns for high urgency and low urgency contacts. Neighbourhoods with more 0 to 4 year old children were associated with more low urgency contacts.

**Table 4:** **Unstandardized regression coefficients for associations of neighbourhood population characteristics and primary out-of-hours contacts** ^**a**^

	**Total contacts**	**Telephone consultations**	**Home visits**	**Consultations**	**High urgency contacts**	**Low urgency contacts**
	**B** ^c^ (95% CI)	**B** ^c^ (95% CI)	**B** ^c^ (95% CI)	**B** ^c^ (95% CI)	**B** ^c^ (95% CI)	**B** ^c^ (95% CI)
Constant^b^	−85.62 (−195.27; 24.02)	−31.39 (−78.86; 16.07)	−16.24 (−34.96; 2.48)	−37.85 (−103.29; 27.59)	−14.42 (−70.81; 41.97)	−70.77 (−136.31; −5.23)
Percentage women	**5.43 (3.45; 7.41)**	**2.70 (1.84; 3.55)**	**0.71 (0.37; 1.04)**	2.04 (0.86; 3.22)	**2.01 (1.01; 3.02)**	**3.43 (2.24; 4.61)**
Age group (percentage) 0–4 years	5.47 (2.38; 8.56)	**1.64 (0.30; 2.98)**	−0.28 (−0.81; 0.25)	**4.12 (2.28; 5.96)**	1.97 (0.41; 3.54)	**4.51 (1.66; 5.36)**
5-14 years	**−2.70 (−3.96; −1.44)**	**−1.99 (−2.53; −1.44)**	−0.35 (−0.56; −0.13)	−0.36 (−1.11; 0.39)	−0.92 (1.56; −0.28	**−1.79 (−2.55; −1.04)**
15-24 years	**−3.58 (−4.74; −2.41)**	**−1.87 (−2.37; −1.36)**	**−0.61 (−0.81; −0.41)**	−1.10 (−1.80; −0.40)	**−2.13 (−2.72; −1.54)**	**−1.46 (−2.16; −0.76)**
40-64 years	0.17 (−0.79; 1.13)	−0.10 (−0.51; 0.32)	−0.09 (−0.26; 0.07)	0.35 (−0.22; 0.92)	−0.15 (−0.64; 0.33)	0.34 (−0.24; 0.91)
65-74 years	−1.61 (−3.22; −0.01)	**−1.50 (−2.19; −0.80)**	**−0.56 (−0.83; −0.29)**	0.44 (−0.52; 1.39)	−0.60 (−1.41; 0.21)	−1.03 (−1.99; −0.07)
75+	1.61 (0.36; 2.86)	0.81 (0.29; 1.35)	**2.01 (1.79; 2.22)**	−1.22 (−1.96; −0.47)	**1.14 (0.51; 1.77)**	0.48 (−0.27; 1.23)
Percentage low-income households	**1.15 (0.62; 1.68)**	**0.52 (0.29; 0.75)**	**0.25 (0.16; 0.34)**	0.39 (0.07; 0.70)	**0.90 (0.64; 1.17)**	0.24 (−0.08; 0.56)
Percentage non-Western immigrants	**0.85 (0.56; 1.14)**	**0.42 (0.30; 0.55)**	0.03 (−0.02; 0.08)	**0.39 (0.21; 0.56)**	**0.34 (0.19; 0.49)**	**0.52 (0.35; 0.70)**
Urbanisation Low urbanisation	12.24 (5.28; 19.21)	**6.20 (3.19; 9.22)**	0.72 (−0.47; 1.91)	5.21 (1.06; 9.37)	5.13 (1.60; 8.67)	6.97 (2.80; 11.14)
Moderate urbanisation	**33.85 (25.53; 42.17)**	**15.00 (11.36; 18.60)**	1.40 (−0.02; 2.83)	**17.33 (12.37; 22.29)**	**13.80 (9.57; 18.02)**	**20.02 (15.04; 25.00)**
High urbanisation	**43.22 (33.97; 52.47)**	**19.37 (15.39; 23.37)**	1.79 (0.21; 3.37)	**21.95 (16.44; 27.47)**	**16.59 (11.89; 21.29)**	**26.73 (21.19; 32.26)**
Very high urbanisation	**23.31 (10.33; 36.29)**	**14.18 (8.56; 19.80)**	−0.76 (−2.98; 1.46)	9.81 (2.07; 17.55)	6.09 (−0.50; 12.69)	**17.53 (9.76; 25.30)**
Neighbourhood status Low status	**27.94 (18.01; 37.88)**	**8.66 (4.36; 12.96)**	2.23 (0.53; 3.93)	**17.02 (11.10; 22.95)**	**10.48 (5.44; 15.53)**	**17.08 (11.13; 23.03)**
Moderate status	7.09 (−0.95; 15.12)	0.93 (−2.55; 4.41)	0.24 (−1.13; 1.61)	5.95 (1.16; 10.73)	0.98 (−3.10; 5.06)	5.89 (1.08; 10.69)
High status	3.77 (−3.58; 11.11)	−0.18 (−3.37; 2.99)	−0.28 (−1.54; 0.98)	4.18 (−0.20; 8.56)	0.62 (−3.11; 4.35)	2.97 (−1.43; 7.36)
**Random effect**	**Variance (SE)**	**Variance (SE)**	**Variance (SE)**	**Variance (SE)**	**Variance (SE)**	**Variance (SE)**
Between GP cooperative variance	1288.30 (421.42)	239.56 (78.22)	29.97 (10.45)	503.30 (173.11)	797.09 (255.70)	394.81 (128.99)
ICC^d^	0.50	0.49	0.44	0.53	0.70	0.46
	**R** ^**2**^	**R** ^**2**^	**R** ^**2**^	**R** ^**2**^	**R** ^**2**^	**R** ^**2**^
Explained variance	22.44	27.32	51.52	10.87	7.98	30.58

^a^Per 1000 inhabitants for postcode areas with > = 1000 inhabitants and > = 100 patients (n = 1121 postcode areas).

^b^Constant: men, age group 20–39, rural area, very high status neighbourhood.

^c^Statistical significance, bold if *P* = <.001.

^d^ICC is the intra-class correlation between GP cooperative catchment areas: the relative contribution of GP cooperative catchment area to the residual variance.

As depicted in Table 5, patterns for the number of primary OOH contacts per disease cluster were similar to patterns for the total number of contacts. However, a somewhat different pattern was found for injuries. Primary OOH contacts for injuries were only significantly associated with low neighbourhood status: i.e. in more deprived neighbourhoods, inhabitants had more primary OOH contacts for injuries. Primary OOH contacts for infections mainly associated with the age group 0 to 4 years old, i.e. in neighbourhoods with more 0 to 4 year old children, more primary OOH contacts for infections took place.

**Table 5:** **Unstandardized regression coefficients for associations of neighbourhood population characteristics and primary out-of-hours contacts** ^**a**^

	**Injuries**	**Infections**	**Long-term health conditions**	**Somatic symptoms and illnesses**	**Psychological and social problems**
	**B** ^c^ (95% CI)	**B** ^c^ (95% CI)	**B** ^c^ (95% CI)	**B** ^c^ (95% CI)	**B** ^c^ (95% CI)
Constant^b^	18.86 (−9.67; 47.39)	−23.98 (−57.58; 9.62)	−19.33 (−46.22; 7.57)	−62.00 (−124.91; 0.84)	−0.80 (−17.47; 15.87)
Percentage women	0.35 (−0.14; 0.85)	**1.04 (0.45; 1.63)**	**0.89 (0.43; 1.36)**	**2.23 (1.14; 3.32)**	0.43 (0.14; 0.72)
Age group (percentage) 0–4 years	0.59 (−0.22; 1.40)	**2.35 (1.40; 3.31)**	0.46 (−0.30; 1.22)	3.11 (1.34; 4.89)	−0.27 (−0.74; 0.21)
5-14 years	0.16 (−0.16; 0.48)	−0.28 (−0.66; 0.09)	−0.53 (−0.83; −0.23)	−0.83 (−1.54; −0.13)	**−0.40 (−0.59; −0.22)**
15-24 years	−0.32 (−0.63; −0.01)	−0.43 (−0.80; −0.07)	**−0.61 (−0.90; −0.32)**	−1.07 (−1.75; −0.39)	**−0.41 (−0.59; −0.22)**
40-64 years	−0.05 (−0.30; 0.21)	−0.03 (−0.33; 0.27)	−0.01 (−0.23; 0.25)	0.15 (−0.41; 0.71)	−0.15 (−0.30; −0.00)
65-74 years	0.02 (−0.39; 0.43)	−0.07 (−0.55; 0.42)	0.08 (−0.31; 0.46)	−0.04 (−0.94; 0.87)	−0.30 (−0.54; −0.06)
75+	−0.13 (−0.45; 0.19)	0.27 (−0.11; 0.65)	**0.66 (0.35; 0.96)**	0.61 (−0.09; 1.32)	0.27 (0.08; 0.45)
Percentage low-income households	−0.03 (−0.16; 0.11)	0.09 (−0.06; 0.25)	0.17 (0.04; 0.29)	0.41 (0.11; 0.70)	0.13 (0.05; 0.20)
Percentage non-Western immigrants	−0.06 (−0.13; 0.02)	**0.26 (0.18; 0.35)**	**0.21 (0.14; 0.28)**	**0.62 (0.45; 0.78)**	0.02 (−0.03; 0.06)
Urbanisation Low urbanisation	0.27 (−2.06; 1.52)	3.49 (1.37; 5.61)	2.48 (0.79; 4.17)	**7.03 (3.09; 10.98)**	1.38 (0.32; 2.43)
Moderate urbanisation	2.12 (−0.12; 4.36)	**8.97 (6.33; 11.62)**	**6.74 (4.63; 8.85)**	**21.33 (16.40; 26.26)**	**4.02 (2.71; 5.33)**
Strong urbanisation	1.51 (−0.88; 3.91)	**11.70 (8.88; 14.53)**	**8.54 (6.29; 10.80)**	**24.52 (19.26; 29.79)**	**4.91 (3.51; 6.32)**
Very strong urbanisation	−2.97 (−6.21; 0.26)	**9.42 (5.60; 13.24)**	4.47 (1.42; 5.08)	**17.86 (10.75; 24.97)**	**4.11 (2.22; 6.01)**
Neighbourhood status Low status	**5.39 (2.80; 7.95)**	1.53 (−1.51; 4.58)	2.66 (0.23;5.08)	8.44 (2.78; 14.11)	1.29 (−0.22; 2.80)
Moderate status	2.65 (0.58; 4.72)	−0.93 (−3.37; 1.52)	−0.18 (−2.13; 1.77)	0.60 (−3.95; 5.15)	−0.11 (−1.33; 1.10)
High status	1.32 (−0.53; 3.18)	−0.94 (−3.14; 1.25)	−1.03 (−2.77; 0.72)	−0.29 (−4.37; 3.79)	−0.15 (−1.24; 0.94)
**Random effect**	**Variance (SE)**	**Variance (SE)**	**Variance (SE)**	**Variance (SE)**	**Variance (SE)**
Between GP cooperative variance	69.50 (28.10)	60.58 (24.74)	68.14 (27.48)	393.27 (158.28)	10.98 (4.56)
ICC^d^	0.60	0.48	0.62	0.64	0.41
	**R** ^**2**^	**R** ^**2**^	**R** ^**2**^	**R** ^**2**^	**R** ^**2**^
Explained variance	14.68	27.15	17.90	11.47	23.10

^a^Per 1000 inhabitants for postcode areas with > = 1000 inhabitants and > = 100 patients. Contacts per disease cluster are calculated using data of 14 GP cooperatives that registered meaningful ICPC-codes for at least 70% of contacts, resulting in data for n = 620 postcode areas.

^b^Constant: men, age group 20–39, rural area, very high status neighbourhood.

^c^Statistical significance, bold if *P* = <.001.

^d^ICC is the intra-class correlation between GP cooperative catchment areas: the relative contribution of GP cooperative catchment area to the residual variance.

The multilevel models showed that sociodemographic characteristics, within the level of a GP cooperative catchment area, partly account for the explained variation in contacts between GP cooperatives. Explained variance (R-squared) of the multilevel models ranged from 8% (high urgency contacts) to 52% (home visits). In addition, multilevel models indicated that the remaining part of the variation in primary OOH contacts between GP cooperative catchment areas, could not be attributed to sociodemographic characteristics of the postcode level. The residual variance, the variance that could not be explained by sociodemographic characteristics, remained high for the GP catchment area level. The intra class correlation (ICC) indicated that 41% (ICC 0.41 for psychological and social problems) to 70% (ICC 0.70 for high urgency contacts) of the residual variance could be attributed to differences between GP cooperative catchment areas. Consequently, the unexplained residual variance resulted from differences between GP cooperatives that were not included in the model.

## Discussion

### Main findings

We found that the overall demand of primary OOH care was higher in neighbourhoods with more female inhabitants, more low-income households, more non-Western immigrants, higher degree of urbanisation level, and low neighbourhood status. Overall, fewer contacts were associated with neighbourhoods with more 5 to 24 year old inhabitants. We found that neighbourhoods with more 0 to 4 year old inhabitants were associated with more (telephone) consultations, low urgency contacts, and contacts for infections. Low neighbourhood status is related to all types of contacts, however, it is no meaningful factor in explaining home visits and diagnoses and symptoms (except for injuries). Infections are mainly related to neighbourhoods with more 0 to 4 year old children, as infections are typically very common among small children [4].

In general, sociodemographic characteristics explain a reasonable proportion of the variation in demand for primary OOH care between GP cooperatives. Especially in explaining the number of home visits, since these are highly related to neighbourhoods with more elderly inhabitants of 75 years and older. However, particularly for GP high urgency contacts, contacts for somatic symptoms and illnesses, and contacts for long-term health conditions, a substantial part of the variance between GP cooperatives can be ascribed to factors other than neighbourhood population characteristics. This indicates that geographical factors may play an important role in these differences (e.g. distance to the GP cooperative, presence of an Emergency Department), given the neighbourhood sociodemographic composition of a GP cooperative catchment area.

Low urgency contacts represented a small majority of the total number of contacts, indicating that almost half of the contacts (48%) took place for (highly) urgent matters. Although the relative proportion of inappropriate use (i.e. presenting low urgent health problems) of primary OOH services is not be as big as it used to be [30], the absolute number of contacts shows an increasing trend [31]. A higher demand of primary OOH care by women and young children is consistent with previous findings [4,12]. The finding that overall demand was higher in low status neighbourhoods, i.e. more deprived areas, is similar to Salisbury [12], who found that patients from more deprived areas contacted a GP cooperative during OOH more frequently than patients from non-deprived areas. Our results show that neighbourhood deprivation was related to (telephone) consultations, however not to home visits. Perhaps in deprived neighbourhoods more patients may have low health literacy skills, and therefore have poorer knowledge of health care services [32], and do not know when to turn to OOH services [33]. Turnbull [34], distinguished between type of contact, and found that neighbourhood deprivation did not affect whether the patient received telephone advice or was seen face-to-face.

Our results also show that part of the differences between GP cooperatives in the number and nature of the primary OOH contacts could not be attributed to sociodemographic composition of the neighbourhood. Various other factors may influence the demand and supply of care [35]. For instance, greater distance of patients to the GP cooperative was found to be associated with lower call rates [36]. Our finding that more variation between GP cooperatives existed for high urgency contacts than low urgency contacts, may indicate differences in substitution of emergency care, or the presence of Emergency Departments near the GP cooperative [8]. For instance, joined triage with Emergency Departments in Integrated Emergency Departments (IED) was found to result in lower utilisation of the ED for low urgent health problems, which instead may be treated by GP cooperatives [37]. In addition, supply-induced demand could be a factor, as more supply of health care may facilitate more health care use [19,38]. Furthermore, the accessibility of the GP during office hours is found to affect patients attending a GP cooperative. E.g. when daytime provision of GP care does not meet the patient’s needs, patient’s may be inclined to use primary OOH care for routine GP care [33,39].

### Strengths and limitations

The present study contributes to the understanding of the association between sociodemographic characteristics and the demand of primary OOH care, based on a large dataset of routinely registered data from electronic health records. Another strength of this study was the use of multilevel models to account for, and assess the scale of the differences between GP cooperative catchment areas. Likelihood-ratio tests indicated that multilevel models fitted the data significantly better than linear regression models. Our findings largely correspond with previous research. To our knowledge, however, the present study is the first to assess the extent to which sociodemographic characteristics explain local demand of primary OOH care. We know now that the contribution of neighbourhood composition varies between type of contacts, and that other factors play an important role in explaining the variation in local demand. An interesting factor to study would be the proximity of an Emergency Department and cooperation between EDs and primary OOH services. However, up-to-date data concerning location, opening hours, and cooperation between EDs and primary OOH services were not available in the period we conducted our analyses.

Since the level of analysis was the postcode level, outcomes of this study should not be attributed to an individual’s demand of OOH care. Due to the ecological fallacy, individual level effects will likely be under- or overestimated when using neighbourhood population characteristics to predict an individual’s behaviour [25]. In addition, since we did not have sociodemographic data on the individual level, we were not able to control for individual effects. Therefore we do not know whether sociodemographic neighbourhood characteristics had an additional effect to individual level characteristics [40]. Furthermore, defining postcode areas as neighbourhood may not reflect the actual neighbourhood patients are living in, as postcode areas are administratively defined and therefore incorrectly can be perceived as an independent community [40]. Nevertheless, since GP cooperative catchment areas are geographically defined by postcode areas, this was not problematic for our analyses. In addition, lower explained variances, R-squared, of population characteristics related to contacts within disease clusters can partly be explained by the smaller sample of GP cooperatives, due to exclusion of GP cooperatives with incomplete registration of ICPC-codes. Finally, registration of ICPC-codes according to version 1 was issued by the Dutch College of General Practitioners (NHG), since version 1 was adapted to use in the Netherlands. Consequently, international comparability of ICPC-codes of the present study is partly limited, since generally ICPC version 2 is used. However, the most common diseases and conditions are comparable between ICPC-2 and the Dutch version of ICPC-1 by the use of sub codes [41].

### Implications for research and practice

The models used for the present study are currently used to predict local demand on four-digit postcode level, for areas where no data about demand of primary OOH care are available. Since sociodemographic characteristics are available for the vast majority of postcode areas in the Netherlands, this method enables estimations for local demand in the greater part of the Netherlands [19]. Sociodemographic characteristics on the neighbourhood level are often relatively easily available and accessible. Combining this sociodemographic information with primary OOH contact data in an estimation model, facilitates a more informed planning of local supply of primary OOH care. For instance, primary OOH services located in low status neighbourhoods, with a substantial portion of elderly inhabitants, may need to allocate more staff and resources than their counterparts based in more affluent neighbourhoods. In addition, some groups may seek less contact with primary OOH services if they are better informed. For instance, in low-status neighbourhoods, increasing health literacy of (part of the) neighbourhood population may facilitate more appropriate use of primary OOH services. Accordingly, the results of this study provide a useful starting point to analyse the demand for primary OOH care based on the sociodemographic composition of neighbourhoods in GP cooperative catchment areas. By which the findings facilitate the improvement of the planning of the supply of primary OOH services. To enable appropriate use of primary OOH services, and assess over- and underserved areas, taking into account the case-mix of an area is a prerequisite.

Nevertheless, more understanding is needed about, for instance, policy factors of a GP cooperative catchment area that are related to the demand for primary OOH care. More research is required to determine to what extent for instance access to GP day-care, triage procedures, presence of Emergency Departments, and joined triage in Integrated Emergency departments affect the number and nature of GP cooperative contacts.

## Conclusions

The sociodemographic composition of the neighbourhood substantially affects the number and nature of primary OOH contacts. Consequently, neighbourhood population characteristics are a fair predictor of the demand of primary OOH care in a specific area and should be taken into account to enable GP cooperatives to better match supply and demand.

## Abbreviations

ED: Emergency Department
GP: General Practice / Practitioner
ICC: Intra Class Correlation
ICPC: International Classification of Primary Care
IED: Integrated Emergency Department
OOH: Out-Of-Hours
NTS: Netherlands Triage System
SES: Socioeconomic Status
